# Novel candidate drugs in anti-tumor necrosis factor refractory Crohn’s diseases: in silico study for drug repositioning

**DOI:** 10.1038/s41598-020-67801-0

**Published:** 2020-07-01

**Authors:** Min Seob Kwak, Hun Hee Lee, Jae Myung Cha, Hyun Phil Shin, Jung Won Jeon, Jin Young Yoon

**Affiliations:** Department of Internal Medicine, Kyung Hee University Hospital at Gangdong, Kyung Hee University College of Medicine, 892 Dongnam-ro, Gandong-gu, Seoul, 05278 Republic of Korea

**Keywords:** Gastroenterology, Inflammatory bowel disease, Crohn's disease

## Abstract

Biologicals like anti-tumor necrosis factor (TNF) therapy for Crohn’s disease (CD) are safe and effective but there is a significant rate of primary and secondary nonresponse in the patients. In this study, we applied a computational approach to discover novel drug therapies for anti-TNF refractory CD in silico. We use a transcriptome dataset (GSE100833) for the anti-TNF refractory CD patients from NCBI GEO. After co-expression analysis, we specifically investigated the extent of protein–protein interactions among genes in clusters based on a protein–protein interaction database, STRING. Pathway analysis was performed using the clEnrich function based on KEGG gene sets. Co-expressed genes in cluster 1, 2, 3, 4, up or down-regulated genes and all differentially expressed genes are highly connected. Among them, cluster 1, which is highly enriched for chemokine signaling, also showed enrichment for cytokine–cytokine receptor interaction and identifies several drugs including cyclosporin with known efficacy in CD. Vorinostat, histone deacetylase inhibitors, and piperlongumine, which is known to have inhibitory effect on activity of NF-κB, were also identified. Some alkaloids were also selected as potential therapeutic drugs. These finding suggest that they might serve as a novel therapeutic option for anti-TNF refractory CD and support the use of public molecular data and computational approaches to discover novel therapeutic options for CD.

## Introduction

Crohn’s disease (CD) involves chronic and progressive transmural inflammation of the bowel characterized by repeated periods of remission and deterioration^1^. Pharmacologic management of CD currently consists of 5-aminosalicylic acid, corticosteroids, purine analogs azathioprine, and 6-mercaptopurine, and biologics including anti-tumor necrosis factor (TNF)-α inhibitors. Although the medical armamentarium continuously expands, some patients remain refractory to current therapeutic strategies.

Biologicals like anti-TNF agents (e.g., infliximab and adalimumab) are safe and effective but there is a significant rate of primary and secondary nonresponse affecting about 36–40% of patients^2–4^. Currently, anti-a4-integrins, natalizumab and vedolizumab, are generally well tolerated, and a therapeutic option available for those patients^5,6^. Another numerous other agents for IBD treatment are currently under investigation, including Janus kinase inhibitors, anti-mucosal vascular address in cell adhesion molecule-1 agents, an anti-SMAD7 antisense oligonucleotide, an anti-interleukin-12/23 monoclonal antibody, and a sphingosine-1-phosphate receptor-1 selective agonist. However, these are limitations that make this treatment not always satisfactory. In addition, other therapeutic options with different mechanisms of action are required. Accordingly, additional novel drugs, which have potentially favorable clinical effects in these patients, are needed.

In this study, we applied a computational approach to discover novel drug therapies for CD in silico using publicly available molecular data measuring gene expression in CD samples and 164 small-molecule drug compounds.

## Results

### Co-expressed genes for intra-cluster interactions

**Figure 1:**
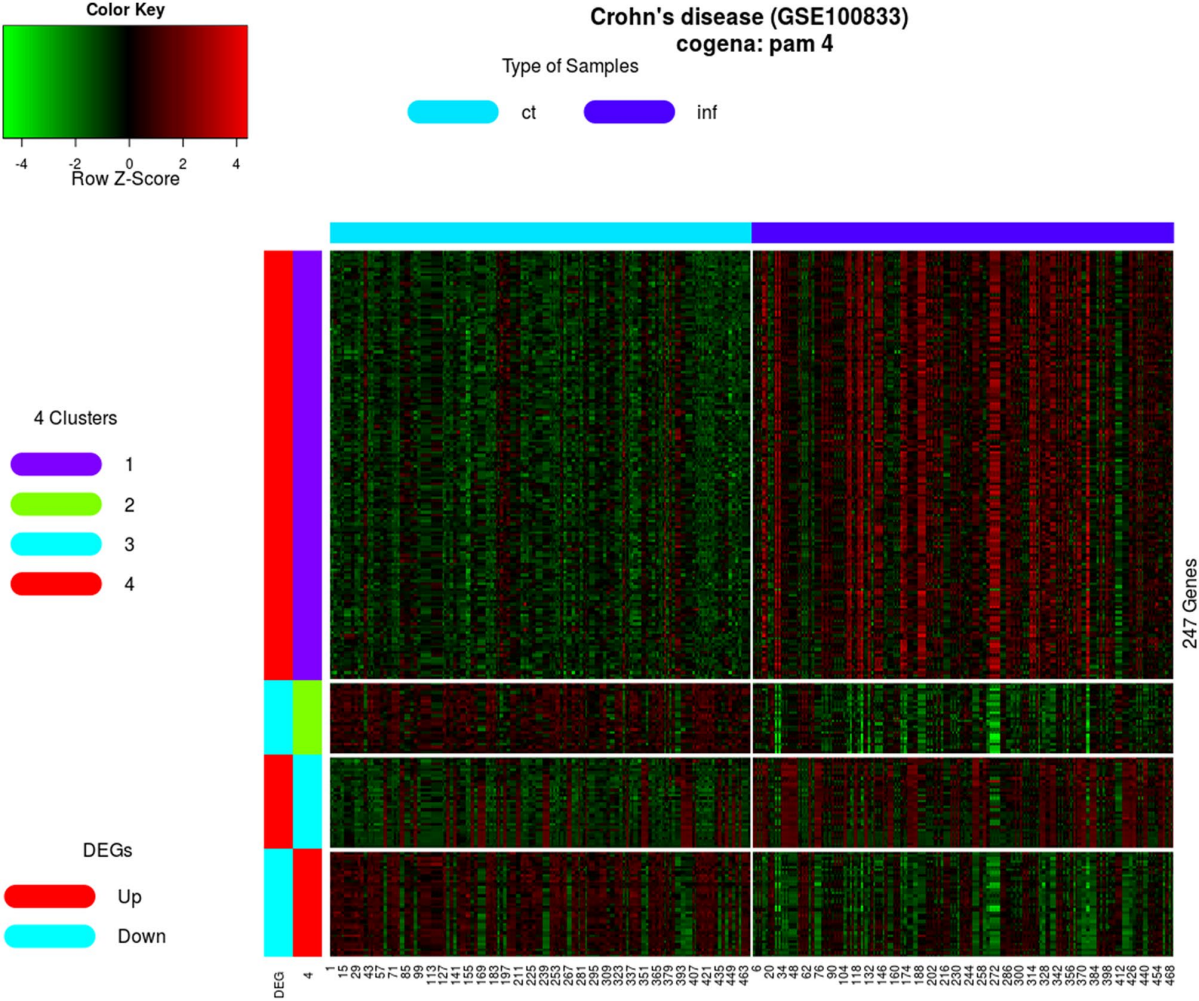
The enrichment scores are shown based on different clusters, up-regulated, down-regulated and DEGs. And the score is correlated with the depth of color. In the x axis, the up-regulated clusters are colored red, while down-regulated clusters are colored green and cluster containing all DEGs is colored blue. The ranked pathways are shown in the y axis used for clusters containing down-regulated genes.

A total of 260 differentially expressed genes (DEGs) were identified (Supplementary Table S1). The consensus clustering algorithm determined an optimal number of four clusters (Fig. 1). The results demonstrate that co-expressed genes in cluster 1, 3, up or down-regulated genes and all DEGs have higher interrelatedness among them and vice versa for other genes clusters (Table 1). Based on the ratio of actual interaction and expected interaction, the connectivity between genes in cluster 1 (with ratio value 4.343) and 3 (with ratio value 9.500), is higher than those in other clusters (Table 1).

**Table 1:** Summary of interactions within clusters for GSE100833.

	The number of genes	The number of protein	Actual interactions	Expected interactions	p-value	Ratio
Cluster 1	158	145	443	102	< 0.001	4.343
Cluster 2	25	25	0	0	1.00	–
Cluster 3	35	31	19	2	< 0.001	9.5
Cluster 4	42	33	3	0	< 0.001	–
Up	193	176	508	136	< 0.001	3.735
Down	67	58	6	2	< 0.001	3
All_DE	260	234	557	178	< 0.001	3.129

STRING interactions are shown for each cluster, up or down-regulated genes and all DEGs, how many genes (gene in cluster), how many proteins (protein in STRING), how many interactions (actual interaction), how many expected interactions (expected interaction), the ratio of actual interactions and expected interactions, together with the p value to get such a number of interactions by chance.

### PPI network and molecular complexes

Perturbed genes that participate big functional clusters are important for studying the mode of action of drugs. A gene/protein interaction network was constructed within each cluster using the STRING (Supplementary Fig. S1). The degree of each node was calculated based on the number of its connections to other nodes and the confidence score cutoff was set as 900 for the analysis. Through Walktrap algorithm analysis, the cluster 1 with the highest degree of association was obtained and 11 subnetworks, including a continent subnetwork, were generated.

### Gene clusters based pathways analysis

**Figure 2:**
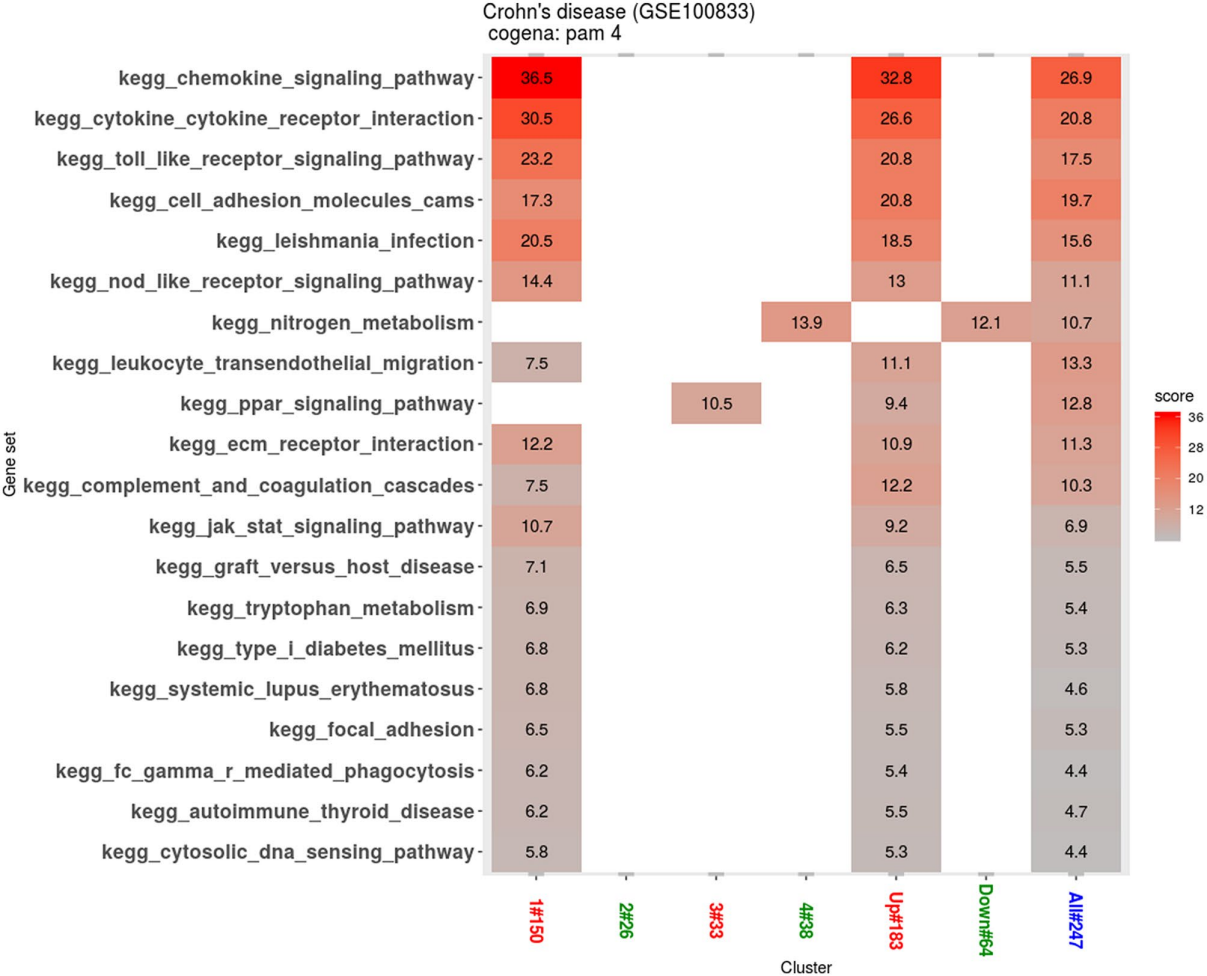
The KEGG pathway analysis results. The enrichment scores are shown based on different clusters, up-regulated, down-regulated and DEGs. In the x axis, the up-regulated clusters are colored red, while down-regulated clusters are coloured green and cluster containing all DEGs is coloured blue. The ranked pathways are shown in the y axis.

To determine the co-expressed genes enrichment in biological pathways, the KEGG analysis was performed on co-expressed gene clusters^7^. It was observed that all the co-expressed genes in all the clusters are highly enriched in inflammatory signaling pathways (Fig. 2). The enrichment scores show that the co-expressed upregulated genes in cluster 1 were highly enriched in chemokine signaling, cytokine–cytokine receptor interaction and toll like receptor signaling pathway (Supplementary Fig. S2), while a small fraction of co-expressed downregulated genes in cluster 4 were enriched in nitrogen metabolism pathways. The upregulated genes in cluster 3 are also observed to be enriched in peroxisome proliferator-activated receptors (PPAR) signaling pathways.

### Drug repositioning analysis

**Figure 3:**
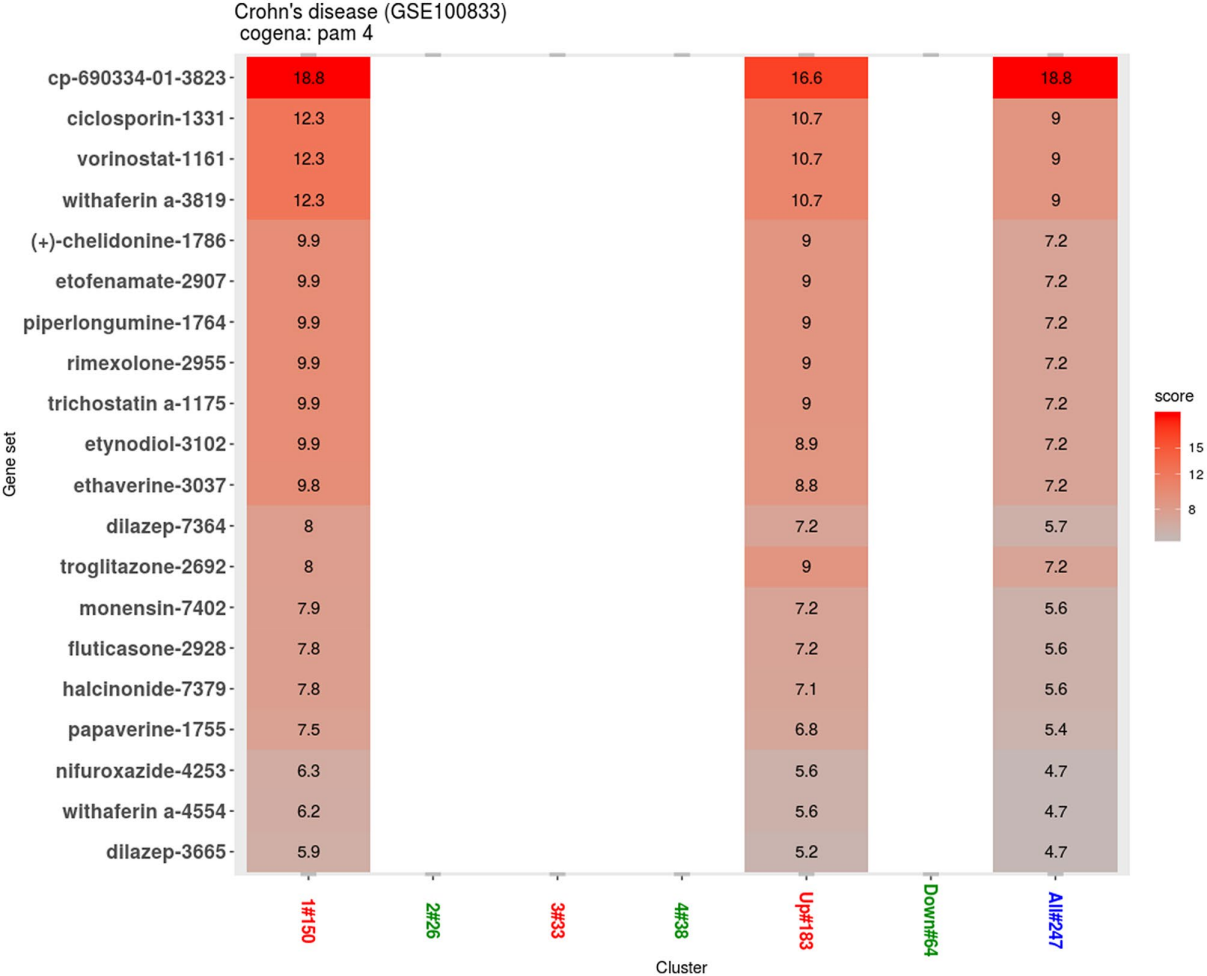
Drug repositioning results based on cluster 1. Enriched drugs with the instance number are shown on the y axis.

**Figure 4:**
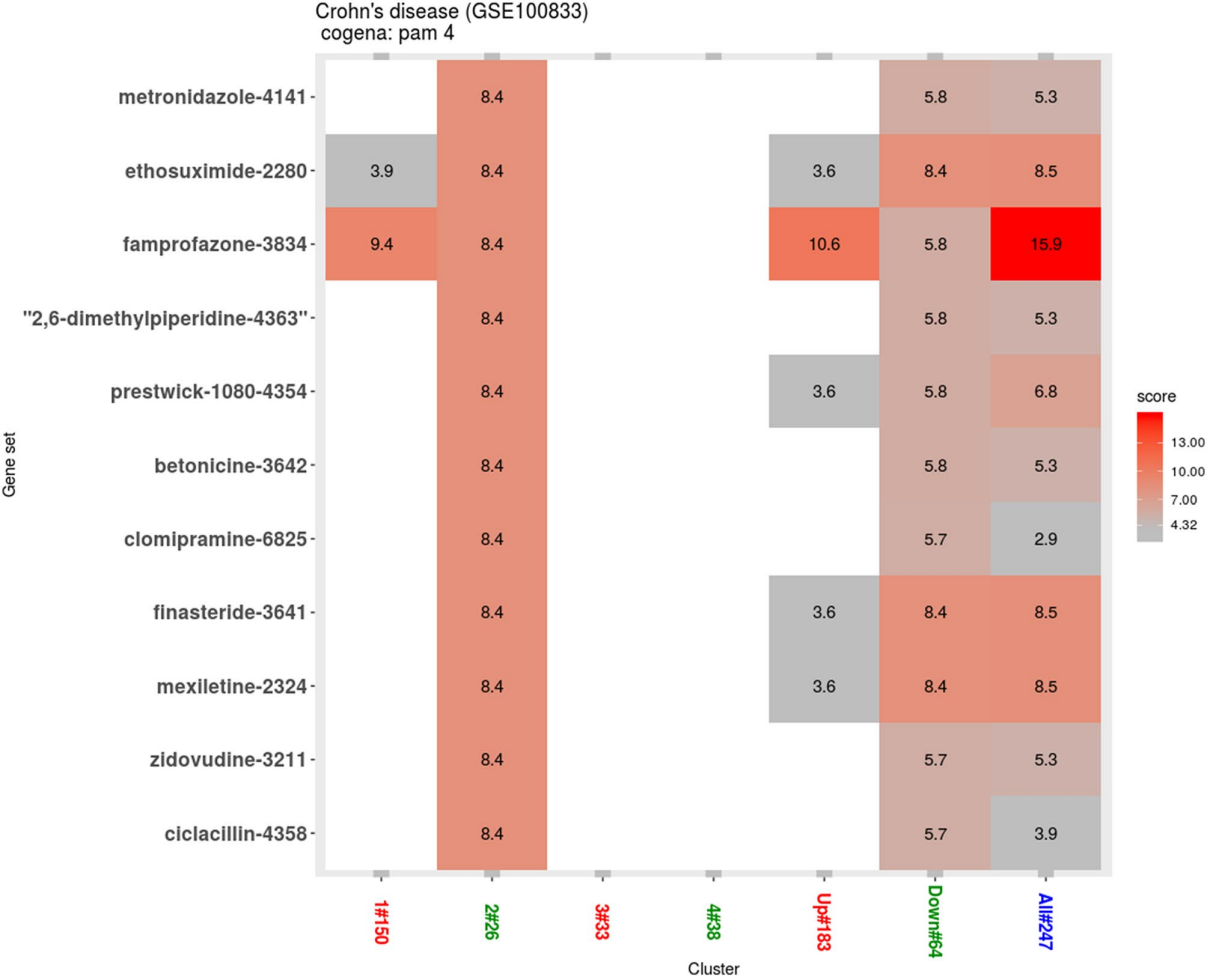
Drug repositioning results based on cluster 2. Enriched drugs with the instance number are shown on the y axis.

The results show a high enrichment score in cluster 1 for CP-690334-01 drug profile which is not available for its information (Fig. 3). Following that, cyclosporine, vorinostat and with aferin drug profiles shows the same and second highest enrichment profiles in cluster 1. (Fig. 3). The drug-response associated genes are shown in Supplementary Fig. S3. CMap drug signatures matching cluster 2 were also investigated (Fig. 4) and 11 drug candidates with same enrichment score of the DEGs were selected as follows: metronidazole, ethosuximide, famprofazone, 2,6-dimethylpiperidine, prestwick, betonicine, clomipramine, finasteride, mexiletine, zidovudine, and ciclacilline.

**Figure 5:**
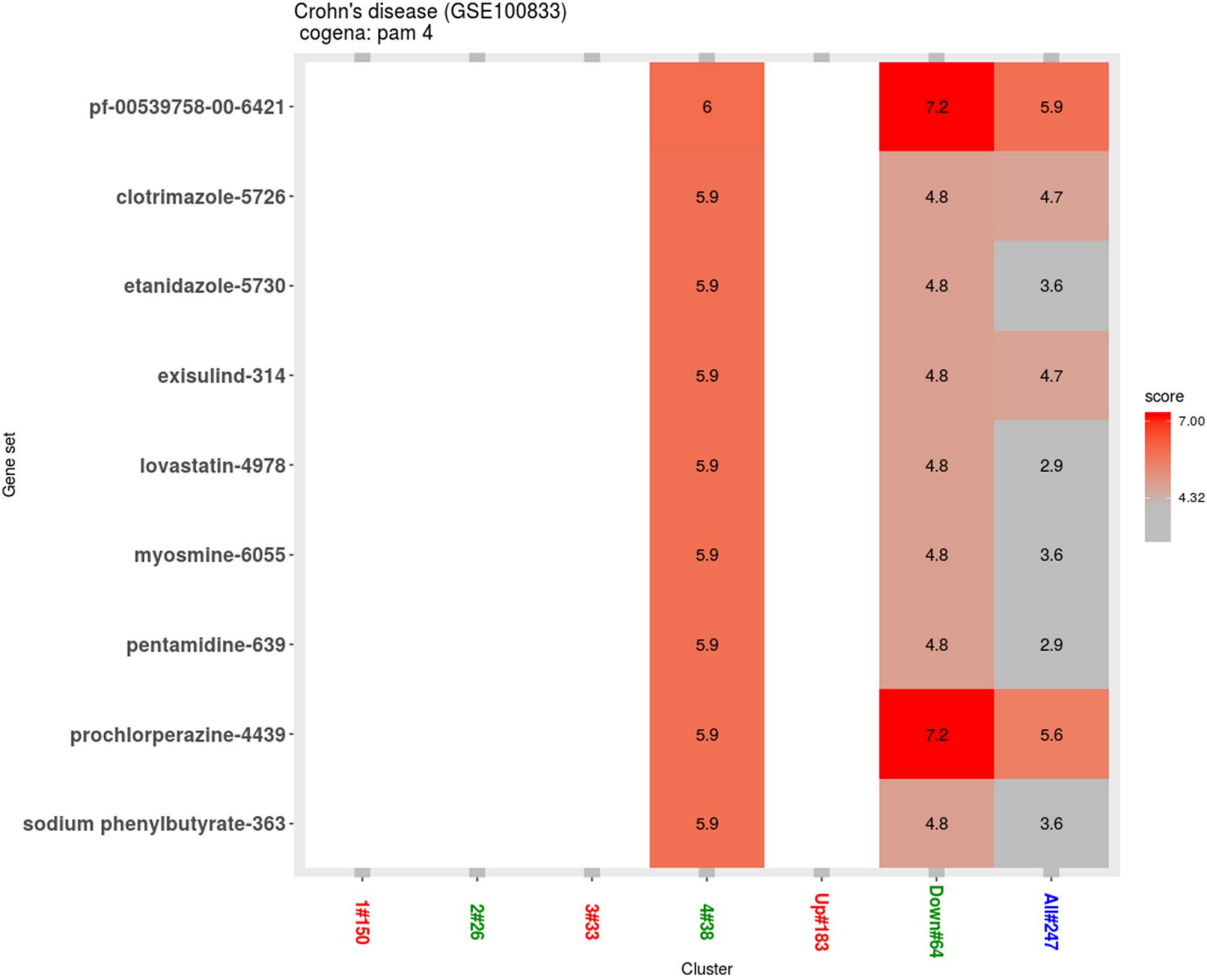
Drug repositioning results based on cluster 4. Enriched drugs with the instance number are shown on the y axis.

Cluster 3 does not show any significant drug profile enrichments, despite of its highly enriched for PPAR signaling pathway. We also observed several candidate drug enrichment profiles (pf-00539758-00, clotrimazole, etanidazole, exisulind, lovastatin, myosmine, pentamidine, prochlorperazine, and sodium phenylbutyrate) in cluster 4, which was enriched in nitrogen metabolism pathway (Fig. 5). However, the ranks of the drugs are relatively lower compared with ranks of drugs in cluster 1 and 2 (Fig. 5).

## Discussion

To the best of our knowledge, this was the first study to predict the drug candidate compounds for anti-TNF refractory CD using CMap-based compound target prediction. Three biologically related groups of pathways, which have an established role in CD, are highlighted in clusters 1 shown in Fig. 2. The chemokine signaling pathway and cytokine–cytokine receptor interaction pathway play important roles in the pathogenesis of CD^8^. Toll-like receptors, as sensors of gut microbiota, play a critical role in maintaining the gut’s homeostasis and controlling the immune responses, thereby inciting CD^9,10^. They have been targeted by biologic drugs for the treatment of CD with some success^11–13^.

We focus our repositioning analysis on cluster 1. Cluster 1, which is highly enriched for chemokine and cytokine receptor interaction, identifies relatively many drug profile enrichments. Also, it is notable that the pathways represented in cluster 1 are now the focus of biologic therapies in CD, perhaps in response to the lack of efficacy of small molecule therapies in these pathways.

The information of CP-690334-01, ranked 1st, is not available, however the overrepresentation in anti-TNF refractory CD patients could give us a clear vision when applied to murine model and real patient. An FDA-approved drug for CD, cyclosporin, is captured and ranked 2nd. The data with respect to the use of cyclosporin in CD are very limited. However, recent Australian prospective study shows that a combination strategy of tacrolimus or cyclosporin with vedolizumab was safe and effective when the induction therapy was introduced to CD patients who had failed vedolizumab monotherapy^14^. This suggests that salvage therapy using cyclosporin may be a strategy to induce remission in to anti-TNF refractory CD patients.

Next, several histone deacetylase inhibitors (HDACi) were identified as potential candidate drugs. Vorinostat are approved for treating certain haematological malignancies, however, recent evidence also illustrates they are modulators of the immune system^15–17^. Therefore, there is emerging evidence that HDACi could have utility in the treatment of rheumatoid arthritis^18–20^, multiple sclerosis^21^, systemic lupus erythematosus^22,23^, and airway hyperresponsiveness^24^, sharing some clinical similarity with CD. There is solid evidence that in in various colitis models in particular, HDACi exert an anti-inflammatory capacity. Several preclinical studies from experimental colitis models reported that the HDACi ameliorated macroscopic and histologic inflammation, in addition to reduced IFN-γ production accompanied by increased production of IL-10^25–27^. Rather, one naturally occurring HDACi, butyrate, decreased TNF production and proinflammatory cytokine mRNA expression by intestinal biopsies and isolated lamina propria cells from CD patients^28^. Trichostatin A, another HDACi, induce anergy in Th cells in vitro, as characterized by an inhibition of proliferation accompanied by a reduction of IL-2 production in Th1 cell cultures^29^. These results that have identified the anti-inflammatory effect of HDACi also support a therapeutic role for CD.

Timely, phase I/II clinical trial is currently recruiting the patients to study the safety and efficacy of vorinostat in treating patients with moderate-to-severe CD who are not controlled by standard maintenance therapy (Clinical Trial ID, NCT03167437). Therefore, providing the efficacy, we expect that novel drug would be available soon.

Corticosteroid, such as withaferin A, rimexolone, etynodiol, fluticasone and halcinonide, is effective treatments for CD, but its systemic use has been widely replaced in the clinic by biologics, due to a shared adverse event risk.

Some alkaloids were also selected as potential therapeutic drugs. Chelidonine has several known therapeutic effects, which include mild analgesic, antimicrobial, and oncostatic effects, and it acts as a sedative in the central nervous system^30^. Further, previous Korean study suggest the chelidonine ameliorates colon injury and inhibits the increase of inflammatory mediators, such as IL-6 and TNF-α, and oxidative damage in murine colitis model^31^. Harris et al.^32^ found that papaverine, another alkaloid, attenuated the disease activity index, in large part by significantly reducing rectal bleeding in DSS-induced colitis model. And there are few studies reporting on the anti-inflammatory activity of ethaverine, the ethyl analogue of papaverine. These results provide novel insights into the pharmacological actions of alkaloid and its potential use for the treatment of intestinal inflammation.

Previous study suggest that piperlongumine, a natural product isolated from the fruit, might modulate reactive oxygen species production under inflammatory conditions for rheumatoid arthritis^33^. The vast majority of literature studies have confirmed beneficial effects of PPAR agonists, such as troglitazone, on macroscopic and histopathological features of colitis^34–36^. However, supporting evidence for therapeutic potential is still limited in remained compounds of cluster 1, compounds enriched for PPAR signaling pathways (cluster 3), and compounds revealed enrichment of nitrogen metabolism pathway (cluster 4).

Several limitations of our study merit discussion. First, biological entities are nonlinear systems showing ‘chaotic behaviour'. As such, there is no relation between the magnitude of the input and the magnitude of the output, with even the most minuscule differences between initial conditions rapidly translating into major differences in the output. And second, there is potentially selection bias from single dataset, which would make the study unrepresentative of the entire anti-TNF refractory CD patients and may restrict the generalizability of our results. Therefore, our computational method would not be able to fully model the complexity of biological systems in CD patients with various confounding factors. Further experiments would therefore need to be performed in multiple, genetically diverse patient samples to confirm this.

In conclusion, among the top compounds predicted to be therapeutic for anti-TNF refractory CD patients by our approach were cyclosporine, a calcineurin inhibitors known to treat IBD, and vorinostat, a HDACi previously described to demonstrate positive results in numerous murine studies. Further, chelidonine and piperlongumine are also identified as potential candidates. These drugs could lead to in-human clinical trials and rapidly and relatively inexpensively offer several new treatments for patients with anti-TNF refractory CD. Since the results are based on in silico analysis, further in-depth studies are necessary to add to the validity of these results.

## Methods

### Data sources and processing

Transcriptomic profiles dataset GSE100833 of anti-TNF refractory CD patients was downloaded from NCBI's Gene Expression Omnibus database (GEO datasets). A total of 327 patients consisting of non-inflamed and inflamed colonic tissues were profiled based on Affymetrix Human Genome U133 Plus 2.0 microarrays platform^37^. Each gene expression profile was normalized using robust multi-array average (RMA)^38,39^ and non-expressed and non-informative genes were filtered using the MetaDE package^40^. The limma package^41^ was used to identify differentially expressed genes with the thresholds of FDR less than 0.05 and absolute logFC more than 1. Co-expression analysis was performed using the coExp function. We have constructed a drug repositioning and drug mechanism-of-action (MoA) discovery pipeline based on the cogena framework using a pathway gene set and the CMap gene set^42^.

### Consensus clustering

We used partitioning around medoids (PAM) clustering based on Euclidean distance, in order to identify the characteristics of gene expression profiles^43^. For the choice of cluster number, ConsensusClusterPlus R-package^44^ was used to identify clusters using 1,000 iterations (reps), 80% sample resampling (pItem) from 2 to 20 clusters (k). The distance matrix was set to Pearson correlation (distance) and linkage function was set as wald. D (innerLinkage) and average (finalLinkag). In order to select optimal cluster number k, we calculated the empirical cumulative distribution (CDF) and the proportional area change under CDF (Δ(k)). According to the Δ(k) vs k plot, the k where Δ(k) started to approach zero was optimal. We also plotted the heatmap of consensus matrix at k to observe whether boundaries of each cluster were sharp. Considering the results of the Δ(k) vs k plot and the heatmap, we determined the optimal cluster numbers.

### Connectivity map query

To find compounds that have similar gene expression patterns, we identified the most significantly (Z < 0.001) up- and down-regulated probes based on log ratio of gene expression of adjacent normal tissue, compared with the inflammatory lesions in anti-TNF refractory CD patients. After removal of duplicates, we ended up with 193 up-regulated genes and 65 down-regulated genes (Supplementary Table S2) that we submitted simultaneously for our CMap query (build02; www.broadinstitute.org/cmap/). Each signature was queried against the CMap using the gene set enrichment analysis algorithm^45,46^. By inputting a gene-expression profile of interest and querying it against the CMap data, a list of ranked CMap drugs is obtained (Supplementary Table S3).

### Protein–protein interactions and pathway analysis

Following co-expression analysis, we specifically investigated the extent of protein–protein interactions among genes in clusters based on a protein–protein interaction database, STRING (Table 1)^47^. The expected interaction and p value are calculated based on a random background model that preserves the degree distribution of the input proteins^47,48^, implemented via the get-summary function in the STRINGdb package. The pathway analysis was performed using the clEnrich function based on KEGG gene sets and visualized with Enrichr program^49,50^. For network analysis, a novel web-based tool, OmicsNet (https://www.omicsnet.ca), was used^51^.

### Drug repositioning based on co-expression analysis

Each of the co-expressed gene clusters were subjected to drug repositioning analysis based on CMap gene sets to identify the likelihood of drug MOA in identified pathways. The statistical analyses were performed using R version 3.5.1^52^. All significant thresholds were set at a two-sided *p*-value of 0.05.

## Supplementary information

Supplementary file1 (PNG 632 kb)

Supplementary file2 (TIF 735 kb)

Supplementary file3 (TIF 783 kb)

Supplementary file4 (DOCX 14 kb)

Supplementary file5 (XLS 64 kb)

Supplementary file6 (XLS 38 kb)

Supplementary file7 (XLS 996 kb)
