# Supplementary figures and images for: Novel candidate drugs in anti-tumor necrosis factor refractory Crohn’s diseases: in silico study for drug repositioning
Source: Sci Rep. 2020 Jul 1;10:10708. doi: 10.1038/s41598-020-67801-0 (PMC7330029; doi:10.1038/s41598-020-67801-0)

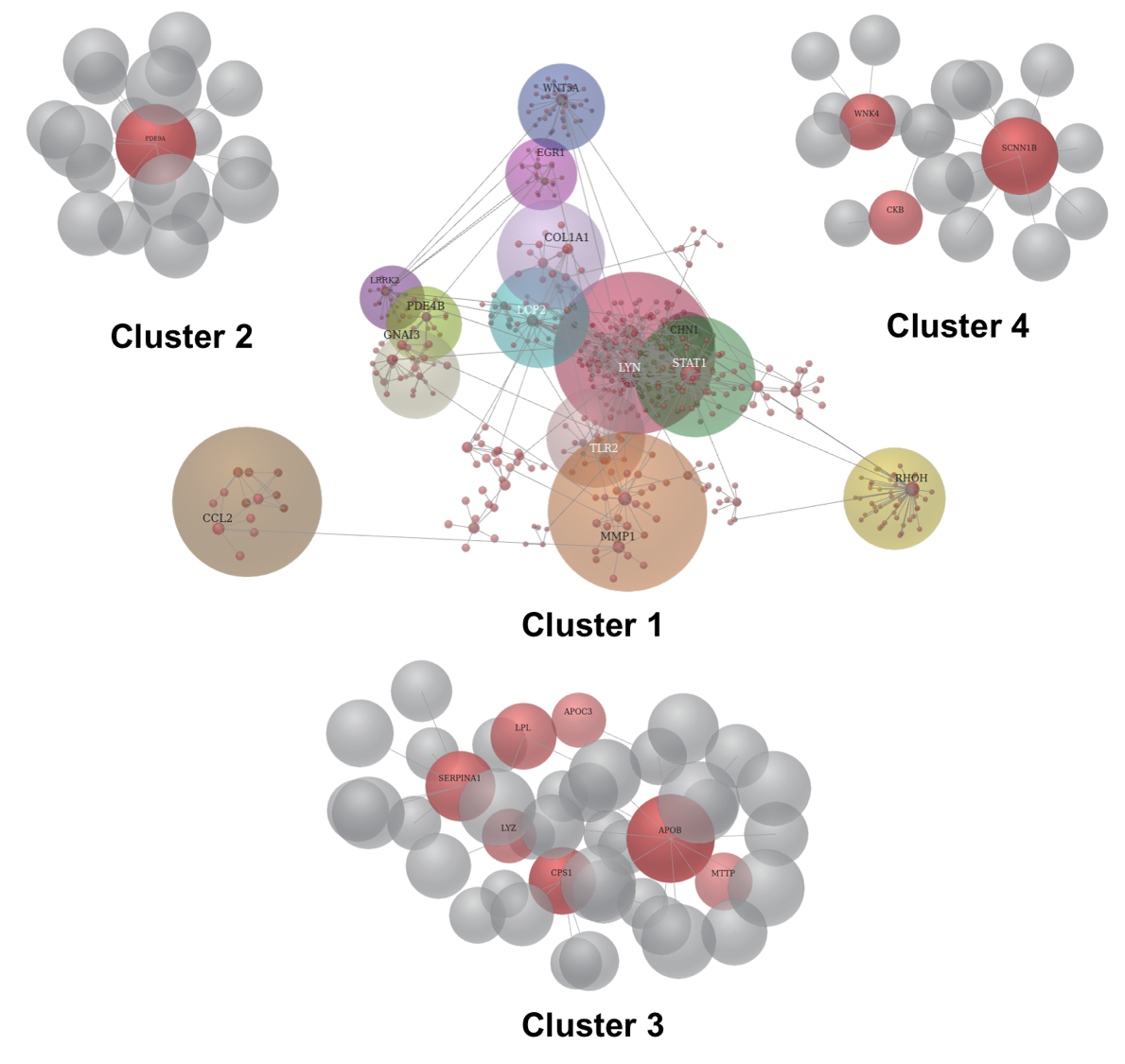

Supplement: Supplementary file 1 — Supplementary file1 (PNG 632 kb) [file 41598_2020_67801_MOESM1_ESM.png]

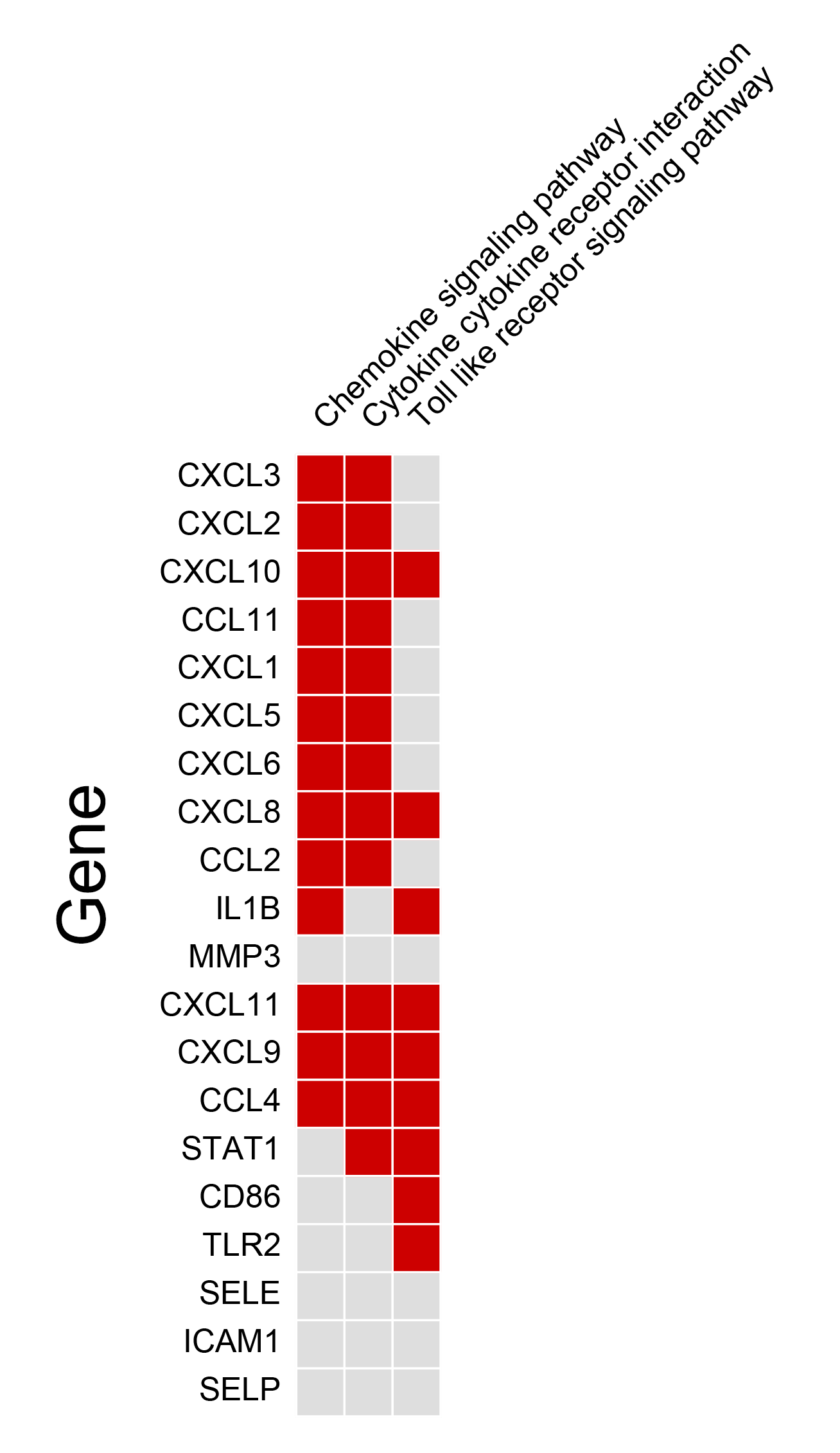

Supplement: Supplementary file 2 — Supplementary file2 (TIF 735 kb) [file 41598_2020_67801_MOESM2_ESM.tif]

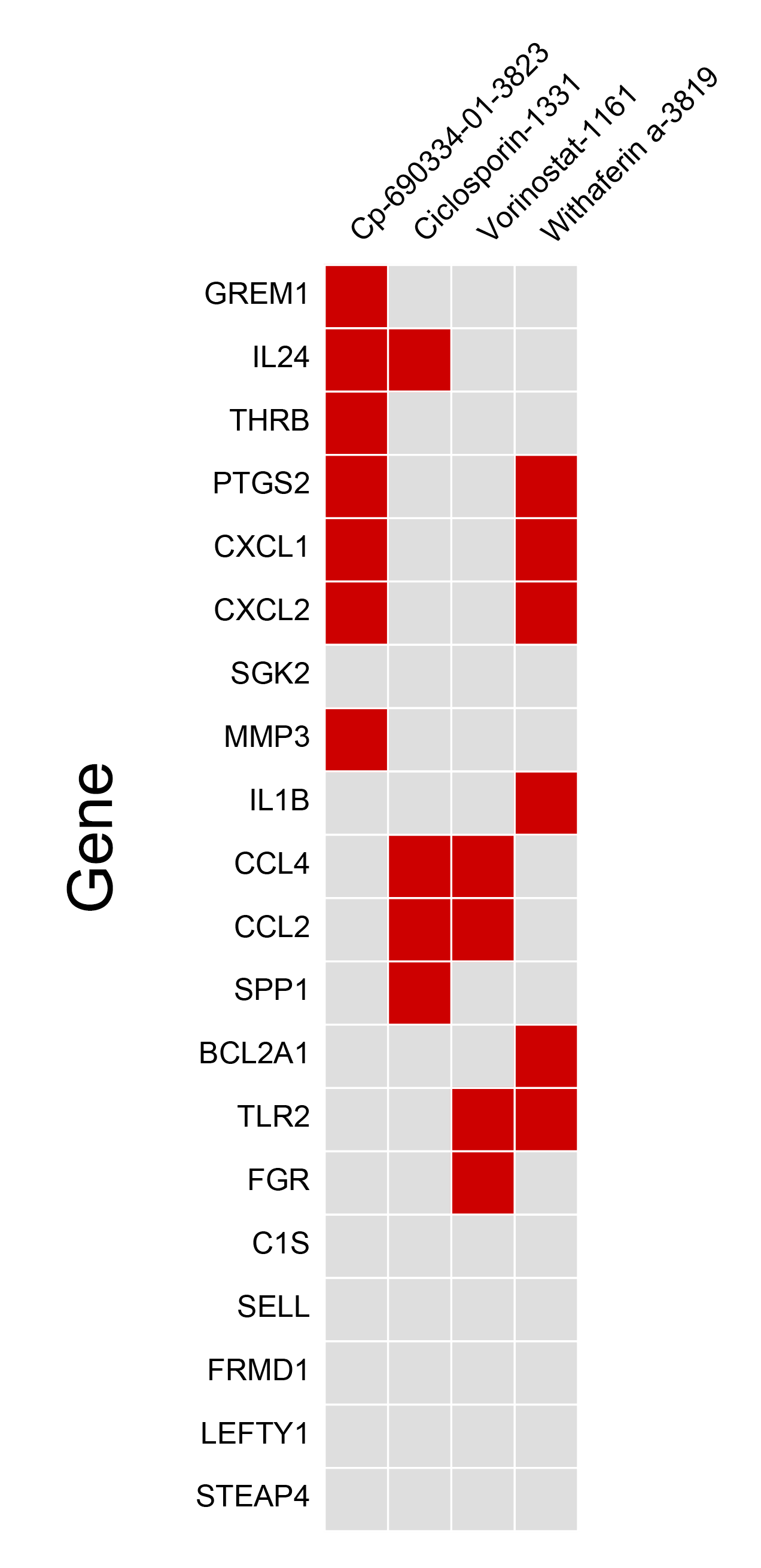

Supplement: Supplementary file 3 — Supplementary file3 (TIF 783 kb) [file 41598_2020_67801_MOESM3_ESM.tif]
