# Supplementary material for: Novel candidate drugs in anti-tumor necrosis factor refractory Crohn’s diseases: in silico study for drug repositioning
Source: Sci Rep. 2020 Jul 1;10:10708. doi: 10.1038/s41598-020-67801-0 (PMC7330029; doi:10.1038/s41598-020-67801-0)
Supplement: Supplementary file 4 — Supplementary file4 (DOCX 14 kb) [file 41598_2020_67801_MOESM4_ESM.docx]

**Novel candidate drugs in** **anti-tumor necrosis factor refractory Crohn’s diseases:** **In silico study for drug repositioning**

**Short title:** Novel drug in Anti-TNFα refractory Crohn’s disease

Min Seob Kwak^1^, Hun Hee Lee^2^, Jae Myung Cha^1^, Hyun Phil Shin^1^, Jung Won Jeon^1^, Jin Young Yoon^1^

^1^Department of Internal Medicine, Kyung Hee University Hospital at Gangdong, Kyung Hee University College of Medicine, Seoul, Republic of Korea

^2^Kyung Hee University Industry-Academic Cooperation Foundation, Seoul, Republic of Korea

**Corresponding Author:** Min Seob Kwak, MD, PhD

Department of Internal Medicine, Kyung Hee University Hospital at Gangdong, Kyung Hee University School of Medicine, 892 Dongnam-ro, Gandong-gu, Seoul 05278, Republic of Korea. Telephone: +82-2-440-6119, Fax: +82-2-440-6295, E-mail address: [kwac63@khu.ac.kr](mailto:kwac63@khu.ac.kr)

**Supplementary Table S1.** The differentially expressed genes between non-inflamed and inflamed colonic tissues in anti-TNF refractory CD patients.

**Supplementary Fig. S1.** The interaction network of differentially expressed genes by Walktrap algorithm. The cluster 1 has a large network consisted of 580 nodes and 806 edges. While, many nodes are not clustered in cluster 2–4, and produced only one small community with 20 ~ 43 nodes. (Cluster 2: 20 nodes and 19 edges, Cluster 3: 43 nodes and 42 edges, Cluster 4: 21 nodes and 20 edges; OmicsNet, https://www.omicsnet.ca)

**Supplementary Fig. S2.** Top 3 KEGG pathways of target genes.

**Supplementary Fig. S3.** Top 4 chemical drugs and their gene targets queried into CMap.
